# A Case of Steroid-Associated Expressive Aphasia

**DOI:** 10.7759/cureus.18863

**Published:** 2021-10-18

**Authors:** Aliza Rizwan, Yechiel S Mor, Allan P Frank

**Affiliations:** 1 Internal Medicine, Detroit Medical Center/Wayne State University, Detroit, USA

**Keywords:** aphasia, steroid, broca’s, reversible, reversible adverse effect

## Abstract

Expressive aphasia (non-fluent aphasia) is characterized by the inability to produce words or sentences. The most common cause of expressive aphasia is stroke, usually due to thrombus or emboli in the middle cerebellar artery or internal carotid artery affecting Broca’s area. We present an important, reversible, and previously undescribed cause of a purely expressive aphasia secondary to steroid use. A case of a steroid-induced expressive aphasia has not yet been described in the medical literature. Recognition of this presentation is critical to appropriate therapy and excess morbidity, particularly as steroid (dexamethasone) utilization has increased since the COVID-19 pandemic.

## Introduction

Expressive aphasia, also known as Broca's aphasia, is characterized by partial loss of the language ability (spoken, manual, or written), while comprehension remains intact. Broca’s aphasia results from injury to the inferior frontal gyrus known as Broca's area. Aphasia affects about two million Americans and is more common than Parkinson’s Disease, cerebral palsy, or muscular dystrophy. Nearly 180,000 Americans acquire the disorder each year. The most common cause of expressive aphasia is stroke, usually due to thrombus or emboli in the middle cerebellar artery or internal carotid artery. Other causes include traumatic brain injury, tumors, brain infections, and degenerative illnesses. We present a previously undescribed, reversible cause of purely expressive aphasia without other focal findings caused by dexamethasone use, a corticosteroid. Corticosteroids are naturally produced by the adrenal gland but synthetic forms of corticosteroids have been used since the 1950s to treat several medical conditions due to their potent anti-inflammatory and immunosuppressive properties. Despite their numerous side effects and spontaneous adverse reactions including those affecting the central nervous system, the use of corticosteroids remains widespread for a large array of clinical conditions, most recently the marked inflammation accompanying COVID-19 infection.

## Case presentation

A 35-year-old female with a history of sickle cell thalassemia and bronchial asthma presented to the emergency department due to new-onset confusion and weakness beginning that afternoon. She also reported uncontrolled generalized sickle-cell type pain for the prior two days. The initial differential for her encephalopathy included hypoxia, metabolic and infectious (meningitis/encephalitis).

On admission the patient was hypotensive with a blood pressure of 88/51 mmHg and hypoxic with oxygen saturation 68% at room air, increasing to 95% on 3L nasal cannula. Significant labs on admission are mentioned in Table 1.

**Table 1:** Labs on admission and discharge. CRP: C-reactive protein; AST: Aspartate transaminase.

Variable	Reference Range	On Admission	On Discharge
White blood cell count	K/CUMM	21.7	9.1
Absolute neutrophil count	K/CUMM	18.4	5.0
Hemoglobin	gm/dl	5.5	7.2
RBC	M/CUMM	1.83	2.45
Platelets	K/CUMM	223	234
Sodium	mMol/L	135	138
Potassium	mMol/L	4.3	4.2
Urea nitrogen (BUN)	7-25 mg/dL	45	13
Creatinine	0.6-1.2 mg/dL	3.88	0.95
Creatine phosphokinase	30-223 units/L	57	26.5
Lactate dehydrogenase	100-240	754	-
D-dimer	<<0.5 mg/L	2.81	-
Ferritin	11-306 ng/mL	1805	-
CRP	mg/L		-
Ammonia	16-53 micromole/L	81	-
AST	13-39 units/L	73	-
T. Bili	<1.5 mg/dL	4.43	-
Pneumococcal antigen	Negative	Positive	-
Covid PCR	Negative	Negative	-

Chest X-ray was negative for acute cardiopulmonary condition. CT brain showed no acute intracranial abnormality; CT venography showed no evidence of cerebral venous thrombosis. Her neuro exam revealed normal speech, showed no deficit other than confusion. She was empirically started on cefepime, vancomycin, and IV dexamethasone 10mg every 6 hours for a working diagnosis of meningitis. She received Lactulose for elevated ammonia. Lumbar puncture returned negative for infectious etiology. Aside from a leukocytosis, she was afebrile with no findings otherwise suggestive of infection or metabolic abnormalities. Empiric antibiotics were de-escalated to ceftriaxone.

The patient's mentation gradually improved: she became alert and oriented to person, place, and time, but was left with an acute expressive aphasia. She spoke incoherently but did demonstrate signs of comprehension. Dexamethasone, initially started due to suspicion of meningitis, was discontinued on day 4. Twenty-four hours post discontinuation her expressive aphasia improved and by 48 hours she had returned to her baseline with full comprehension and speaking abilities. She was discharged home after complete resolution of the expressive aphasia, attributed to dexamethasone use. The patient did not get MRI brain done on discharge, although MRI brain several months post discharge was unremarkable. Labs on discharge are mentioned in Table 1.

A transient localized neurological deficit (expressive aphasia) could be associated with high-dose steroid use in our patient with sickle-cell thalassemia and bronchial asthma. Focal neurological symptoms began after dexamethasone use and resolved 48hr after its discontinuation, thus suggesting a possible causal relationship. Several case reports link steroid use to central nervous system (CNS) arterial occlusion (retinal artery, corpus callosum, basal arteries, etc.) and the association between thromboembolism and oral contraceptives is also well known in the medical literature. However, there is no previous literature describing a temporal lobe alteration associated with steroid use.

## Discussion

Corticosteroids with anti-inflammatory or anti-asthmatic actions are synthetic analogs of the natural adrenal cortical hormone hydrocortisone (cortisol). Various modifications in the molecular structure of hydrocortisone influence the intrinsic potency of the steroids and alter some of their pharmacokinetic properties [1].

The use of dexamethasone is widespread amongst a broad spectrum of clinical conditions which benefit from its immunosuppressive and anti-inflammatory effects, such as chronic inflammatory lung disease, various dermatomal disorders, allergic reactions, treating several retinal diseases, for the acute treatment of CNS trauma/adjunct treatment for disease, bowel disorders, arthritis, immune system disorders, blood/hormone disorders, and cancer. Dexamethasone has been a staple of neurology and neurosurgical treatment for over half a century. In the context of malignant brain tumors, it is used to control peri-tumoral edema and to alleviate symptoms of high intracranial pressure or focal neurologic symptoms.

There are various factors that influence both the therapeutic and adverse effects of glucocorticoids including: dosing, time of the doses, duration of treatment, biological potency, metabolism, and pharmacokinetic properties of the glucocorticoid. Corticosteroids act mainly via three principal mechanisms of action: 1) inducing the expression of anti-inflammatory proteins by IkB and MAPK phosphatase I, 2) inhibiting the synthesis of inflammatory proteins blocking NF-kB, and 3) inhibiting 5-lipoxygenease and cyclooxygenase-2 [2].

Although dexamethasone has a wide range of uses it has been associated with a variety of adverse events including muscular weakness, hyperglycemia, cushingoid symptoms, mental disorders, and gastrointestinal ulceration [3]. Steroid use is also associated with blood viscosity, RBC column buildup, vasoconstriction, etc. As demonstrated in an in vivo study - The influence of corticosteroids on human erythropoiesis - findings complement the results of in vitro studies, indicating that erythropoiesis in normal human bone marrow is stimulated by corticosteroid therapy [4]. Marks et al. reported steroid-induced vasoconstriction: glucocorticoid antagonist studies demonstrated the vasoconstrictor effects of topical steroids are mediated by occupancy of classical glucocorticoid receptors, rather than by nonspecific pharmacological mechanisms [5].

The use of corticosteroids has previously been associated with the development of neurological/psychiatric side effects, due to the wide expression of glucocorticoid receptor (GR) in the brain leading to functional and anatomical alterations due to their long-term modulation [6]. Encephalopathy with neurotoxicity can have various manifestations with ranging severities. Mild encephalopathy is characterized by impaired attention, disorientation, short-term memory with preserved alertness. Whereas, severe neurotoxicity often began as mild somnolence, disorientation, impaired attention, and difficulty naming before progressing to global aphasia, myoclonus, depressed level of consciousness, encephalopathy, and seizures [7].

Localized CNS effects due to specific arterial occlusions are associated with steroid use, as demonstrated in the case of central retinal artery occlusion associated with steroid injection for a periocular juvenile hemangioma [8]. Another case reports of quadriparesis and brainstem herniation after selective cervical transforaminal block; a potential role for corticosteroid particulate embolus leading during unintended intra-arterial injection as a potential mechanism [9]. Deleterious effects of intra-arterial administration of particulate steroids on microvascular perfusion in a mouse model have also been reported. Several particulate steroids have an immediate and massive effect on microvascular perfusion because of the formation of RBC aggregates associated with the transformation of RBCs into spiculated RBCs [10]. This supports the idea of specific vascular occlusion rather than generalized brain alteration.

We reasoned corticosteroids induce RBC column buildup, milliary emboli, and vasoconstriction leading to localized obstructive encephalopathy that may have caused expressive aphasia (characterized by partial loss of the ability to produce spoken, manual, or written language with preserved comprehension) in our patient occurred as a rare adverse reaction to dexamethasone with complete resolution upon its discontinuation. The patient had several other risk factors for localized obstructive encephalopathy such as sickle cell anemia + thalassemia (chronic anemia + tissular hypoxia). Bronchial asthma (chronic hypoxemia, even though no acute lung alterations were discovered), leukocytosis (hypercoagulability/viscosity, elevated O2 consumption by WBC worsening hypoxia), hyperammonemia, and elevated BUN also increase osmolality/viscosity. While ammonia may cause encephalopathy by itself, it is usually in the higher range - the patient only had mild hyperammonemia (64 mmol/L) but elevated BUN (45mg/dl) on admission. Polycythemia and thrombocytosis, both increase the viscosity - the patient's RBC and platelets were within normal limits. Several articles associate local corticosteroid use (facial/cranial injections) to local arterial occlusion so it is definitely a possibility, considering risk factors for hyperviscosity. There are several case reports linking steroid use to retinal artery occlusion, cerebral infarction (corpus callosum), and other vascular complications [4,5]. Some patients in case reports are known for chronic conditions, often autoimmune or vascular (alopecia areata, vitiligo, sickle cell, asthma). Other reports are known for anabolic steroid abuse. This is a rare case, unreported in the literature to date, that we encountered. However, generalized encephalopathy would have also had generalized manifestations. Expressive aphasia was the only sign in this case. Risk factors seem to be related to altered blood viscosity or dosage abuse. Further investigational studies are warranted to determine the correlation of dosage and duration of steroid use on this observed side effect.

## Conclusions

We described a rare case of reversible expressive aphasia associated with steroid use. As steroids continue to be incorporated in the medical management of a variety of pathological conditions, including COVID-19 infection, knowing hyperviscosity risk factors, steroid-associated thrombi formation, and dose-dependent vascular adverse effects to possible vascular complications, both central or peripheral is paramount to properly inform patient management. Further investigational studies are warranted to determine the correlation of dosage and duration of steroid use on this observed side effect. Also, whether the addition of high-dose anticoagulant agents such as rivaroxaban/clopidogrel upon admission in high-risk population (blood viscosity increased due to infection, hypoxia, leukocytosis/polycythemia/thrombocytosis, diabetes, hyperammonemia, etc.) who need steroids for optimum management would outweigh the risk of vascular complication.
